# Hyponatraemia in Neck of Femur Fracture: A Narrative Review of Epidemiology, Pathophysiology, and Outcomes

**DOI:** 10.3390/geriatrics11040085

**Published:** 2026-07-13

**Authors:** Amirmohammad Heidari, Kiana Heidary, Hussain Aladdin Leelo, Mohamed H. Ahmed

**Affiliations:** 1 Royal Stoke Hospital, University Hospitals North Midlands, Stoke-on-Trent ST4 6QG, UK; 2 Medical School, University of Nicosia, Nicosia 2417, Cyprus; heidary.k@live.unic.ac.cy (K.H.); leelo.h@live.unic.ac.cy (H.A.L.); 3 Department of Medicine for Older People, North Cheshire and Mersey NHS Foundation Trust, Warrington WA5 1QG, UK; 4 Department of Diabetes, North Cheshire and Mersey NHS Foundation Trust, Warrington WA5 1QG, UK; 5 Faculty of Medicine and Health Sciences, University of Buckingham, Buckingham MK18 1EG, UK

**Keywords:** fracture neck of femur, hyponatraemia, operative period

## Abstract

**Background:** Hyponatraemia is the commonest electrolyte disturbance encountered in older adults admitted with neck of femur (NOF) fracture. It is now recognised both as associated with fragility fracture and as an independent prognostic indicator for adverse post-operative outcomes. **Methods:** Narrative review of the literature, with emphasis on cohort studies, meta-analyses and mechanistic investigations pertinent to hip fracture in adults. **Results:** Admission hyponatraemia affects approximately 13–20% of NOF patients, twice the prevalence observed in age-matched community-dwelling elders and broadly comparable to general geriatric inpatients. A further 20–30% develop in-hospital, predominantly post-operative, hyponatraemia. Mild hyponatraemia (130–135 mmol/L) accounts for 75–85% of cases. Pathophysiology is multifactorial: hypovolaemia from the fracture haematoma, fasting and pre-admission “long lie”; drug effects (thiazides, selective serotonin reuptake inhibitors (SSRIs), proton pump inhibitors, carbamazepine, opioids); and non-osmotic arginine vasopressin (AVP) release driven by pain, nausea and peri-operative stress. Chronic hyponatraemia is hypothesised to contribute to fracture risk through three convergent mechanisms, direct sodium-dependent stimulation of osteoclastogenesis with AVP-mediated bone resorption, subtle cerebral dysfunction producing gait and attention deficits, and sarcopenia, although much of this mechanistic evidence derives from animal and in vitro studies rather than from patients with hip fracture. Hyponatraemia is reproducibly associated with longer length of stay, delayed surgery, and an adjusted 30-day mortality hazard of approximately 1.15–1.40. A dose–response relationship with severity is demonstrable; pre-operative correction has not been shown to improve outcomes in any randomised trial. **Conclusions:** Hyponatraemia in NOF fracture is consistently a consequence of the acute event and, at minimum, a robust marker of frailty and adverse prognosis. Whether it also causally contributes to fracture risk remains unproven, since the supporting human evidence is entirely observational and mechanistic, each contributing study carries methodological weaknesses that warrant caution, and no interventional study has established causality. Where hyponatraemia is mild and isolated, current evidence does not support delaying surgery; moderate and severe hyponatraemia warrant individualised assessment, with cautious correction proceeding alongside surgical planning rather than postponing it. Given the absence of interventional evidence, no correction strategy can yet be recommended to improve fracture or surgical outcomes. Prospective trials of targeted correction strategies and rehabilitation outcomes are overdue.

## 1. Introduction

Hip fracture remains a defining event of old age, affecting an estimated 1.6 million people worldwide annually, and in the United Kingdom around 70,000 patients are admitted each year with a fragility fracture of the proximal femur. Despite iterative improvements in perioperative care captured by the National Hip Fracture Database (NHFD), 30-day mortality persists between 6 and 8% and one-year mortality around 25–30%. Attention has therefore shifted towards modifiable medical cofactors, among which hyponatraemia has emerged as a plausible biomarker and potential target [1,2].

Once dismissed as an incidental finding of the frail elderly, hyponatraemia is now recognised as both independently associated with prevalent fracture and as a marker of adverse peri-operative outcome. The picture is complicated because hyponatraemia in NOF patients represents a bidirectional phenomenon: chronic mild hyponatraemia predates and is associated with falls and fracture, while the fracture event itself, with its attendant pain, immobility, concealed blood loss, drug exposures, fasting and surgical stress, precipitates further incident dysnatraemia. This dual identity has profound implications for clinical reasoning, prognostic counselling and the contentious question of whether to delay surgery for correction [3,4].

Importantly, hyponatraemia is the commonest electrolyte disturbance in hospitalised adults, affecting 15–30% of unselected inpatients [5,6]. Its prevalence is markedly higher in older surgical populations, where polypharmacy, blunted thirst, age-related decline in renal concentrating capacity and accumulating comorbidity all conspire to disturb sodium homeostasis [7]. In adults presenting with NOF fracture specifically, contemporary cohort studies converge on an admission prevalence of approximately 13–20%, with outlier estimates ranging from 2.8% to 26.5% according to threshold definition. The Danish national registry of 7317 patients reported 19.0% [8], a Tayside cohort of 3897 patients reported 19.1% [9], and the Aberdeen prospective fragility fracture cohort of Cumming et al. [3] documented 13.4% on admission, with a further 12.6% acquiring hyponatraemia during the inpatient stay. Because the original report presents these as separate figures without specifying the overlap between the two groups, their sum represents an upper-bound estimate of cumulative in-admission exposure (≈26%, assuming no patient appears in both groups). Italian, Japanese and US case–control series complete a narrow distribution of estimates [10,11,12,13,14]. The 2018 meta-analysis by Corona and colleagues, pooling 15 studies and 51,879 patients, derived a Mantel–Haenszel odds ratio of 2.00 (95% CI 1.43–2.81) for hip fracture in hyponatraemic compared with normonatraemic individuals [15].

Incident in-hospital, predominantly post-operative, hyponatraemia is the more preventable phenotype. Rudge and Kim [16] reported a 27% (95% CI 21.7–32.5) incidence of moderate post-operative hyponatraemia in 254 operated traumatic hip fractures, with a mean post-operative sodium fall of 1.8 mmol/L; Tinning et al. [9] observed hyponatraemia in 29.5% of patients during the first 24 post-operative hours. Across studies, acquired hyponatraemia rates cluster at 20–30% using a <135 mmol/L threshold, with severe cases (<125 mmol/L) uncommon (<1–3%). Severity distribution within NOF cohorts is consistent and skewed toward mild disease: Cumming et al. [3] reported that 75.8% of hyponatraemic fragility fracture patients had mild disease (130–135 mmol/L); a comparable elective arthroplasty cohort showed 81.6% mild, 17.1% moderate and 1.4% severe [17]. For context, the NHANES-derived weighted prevalence of hyponatraemia in the general US adult population is 1.72% [18], rising to 7–8% in community-dwelling elders [19] and approximately 22–23% on geriatric wards [6]. The roughly two- to ten-fold elevation of admission hyponatraemia prevalence in NOF fracture compared with the community reflects the characterisation of hip fracture patients as an especially frail, polypharmaceutically treated and dehydrated subgroup.

The clinical significance of low sodium in NOF patients extends beyond a laboratory abnormality. Even mild chronic hyponatraemia can be associated in some individuals with gait instability, attention deficits and a roughly doubled fall risk in ambulatory older adults [20,21]. Independently of falls, sustained low sodium is associated with accelerated bone resorption and reduced bone mineral density [2,22]. Within the peri-fracture pathway, hyponatraemia is reproducibly associated with longer length of stay, delayed surgery and increased mortality, with adjusted 30-day mortality hazard ratios of approximately 1.15–1.40 across major cohorts [8,9,23].

This narrative review integrates epidemiological, pathophysiological and outcome data relevant to NOF fracture.

## 2. Literature Sources

This is a narrative review. We drew on the published literature on hyponatraemia and hip fracture in older adults, together with the reference lists of retrieved articles and earlier reviews. Cohort studies, meta-analyses and mechanistic investigations were prioritised, and adjacent populations, including fragility fracture, elective hip arthroplasty and hospitalised older adults, were drawn on where data specific to neck of femur fracture were sparse. Numerical estimates are reported as given in the primary sources. Because the sodium thresholds, outcome definitions and degree of covariate adjustment differ substantially between studies, the findings are described and compared narratively rather than pooled. The individual studies are appraised descriptively, and the specific methodological weakness of each principal study, together with the reason it calls for cautious interpretation, is set out in the critical appraisal in Section 6.

### 2.1. Prevalence, Type and Risk Factors for Hyponatreamia

Across contemporary cohort studies, admission hyponatraemia affects 13–20% of hip fracture patients, with outlier estimates spanning 2.8% to 26.5%, largely attributable to variable sodium thresholds. The Danish national registry study by Madsen and colleagues (*n* = 7317 patients ≥ 60 years) reported a prevalence of 19.0% at a threshold of <135 mmol/L [8]. A large Scottish cohort from Tayside (*n* = 3897) yielded almost identical figures of 19.1% at admission [9]. In Aberdeen, Cumming et al. [3] prospectively documented 13.4% admission prevalence in an elderly fragility fracture cohort of whom just over half had a proximal femoral fracture, with an additional 12.6% acquiring hyponatraemia in hospital. As these are reported separately and the degree of overlap between admission and incident cases is not specified, the summed figure (≈26%) should be read as an upper-bound estimate of cumulative in-admission exposure rather than a directly measured prevalence. Italian ([10]: 19.5%; [11]: 9.4% on admission for intracapsular fracture, rising to 18–22% by sex and fracture-type subgroup when defined as ≥one episode during the inpatient stay), Japanese ([12]: 9.6%), and US case–control data ([13]: 9.1%; [14]: 16.9%) complete a broadly comparable distribution. The 2018 meta-analysis by Corona and colleagues, pooling 15 studies and 51,879 patients, yielded a Mantel–Haenszel odds ratio of 2.00 (95% CI 1.43–2.81) for hip fracture in hyponatraemic compared with normonatraemic individuals [15].

Incident in-hospital hyponatraemia is arguably the more preventable phenotype. Rudge and Kim [16], in a single-centre UK series of 254 operated traumatic hip fractures, reported a 27% (95% CI 21.7–32.5) incidence of moderate post-operative hyponatraemia (<135 mmol/L) and 9% severe (<130 mmol/L), with a mean post-operative sodium fall of 1.8 mmol/L. Tinning et al. [9] observed hyponatraemia in 29.5% of patients in the first 24 post-operative hours, declining to 20% at discharge. Across studies, the acquired hyponatraemia rate clusters at 20–30% using a <135 mmol/L threshold, with severe cases (<125 mmol/L) uncommon (<1–3%) [24].

The NHANES-derived weighted prevalence of hyponatraemia in US adults is 1.72%, rising to approximately 7–8% in community-dwelling elders in the Rotterdam population [18,19]. Terzian and colleagues [25] documented 3.5% on admission in general geriatric inpatients. By contrast, a systematic review of hyponatraemia on geriatric wards by Mannesse et al. [6] derived a pooled prevalence of approximately 22–23%, higher than that in other inpatient settings and comparable to the NOF experience. The roughly two- to ten-fold elevation of admission hyponatraemia prevalence in NOF compared with the community is therefore consistent with the characterisation of hip fracture patients as an especially frail, polypharmaceutically treated and dehydrated subgroup.

Although severity classification nomenclature varies (mild 130–135, moderate 125–129, severe < 125 mmol/L per the 2014 European guideline [1]), distribution within NOF cohorts is consistent. Cumming et al. [3] reported that 75.8% of hyponatraemic fragility fracture patients had mild disease. In the elective arthroplasty cohort of Cunningham et al. [17], closely analogous in demographics, mild hyponatraemia accounted for 81.6% of cases, moderate 17.1% and severe 1.4%. A large Chinese hip fracture series of 1001 patients (overall hyponatraemia prevalence 12.6%, 126/1001) reported a closely concordant distribution once expressed as proportions of hyponatraemic patients: mild 77.8% (98/126), moderate 14.3% (18/126) and severe 7.9% (10/126) [26]; the corresponding whole-cohort figures were 9.8%, 1.8% and 1.0%, respectively. Expressed on a like-for-like basis (proportion of hyponatraemic cases), all three cohorts therefore agree that the clinical majority of NOF hyponatraemia is mild, and clinically asymptomatic in the conventional sense.

### 2.2. Risk Factors

Consistent across cohorts are advanced age, female sex ([9]: 78%; [3]: 78%), frailty, polypharmacy and hypovolaemia. Dehydration is documented as contributory in approximately 70% of hyponatraemic fragility fracture cases [3]. Specific drug associations include thiazide diuretics, present in up to 76% of hyponatraemic patients [3], with bendroflumethiazide a dominant agent in UK series; selective serotonin reuptake inhibitors (SSRIs) and related antidepressants (mirtazapine, venlafaxine); proton pump inhibitors (PPIs), now recognised as independent risk factors in a large Swedish case–control study, in which newly initiated PPI therapy was associated with increased odds of hospitalisation due to hyponatraemia [27] (the original odds-ratio estimate was subsequently revised in a 2021 corrigendum); carbamazepine and oxcarbazepine through potentiation of vasopressin action; and opioids, almost universal in peri-operative NOF care.

Overrepresented comorbidities include heart failure, cirrhosis, chronic kidney disease, diabetes mellitus (22.2% in hyponatraemic versus 10.3% in normonatraemic Rotterdam participants), malignancy, hypothyroidism and adrenal insufficiency, though the last two are rarely quantified in NOF-specific cohorts.

## 3. Direction of the Association: Antecedent or Consequence?

The observational evidence is consistent with both directions. Several prospective studies report chronic mild hyponatraemia as an antecedent predictor of fragility fracture, although their observational design cannot establish causation. In the Rotterdam study (*n* = 5208), Hoorn and colleagues reported a hazard ratio for non-vertebral fracture of 1.39 (95% CI 1.11–1.73), which persisted after adjustment for bone mineral density (BMD) and falls [2]. The MrOS study of 5122 community-dwelling men demonstrated analogous associations [28]. Ayus and colleagues [29], examining 31,527 older adults, derived an adjusted hazard ratio for hip fracture of 4.52 (95% CI 2.14–9.60) in those with chronic hyponatraemia sustained over ≥90 days, rising to 7.61 for sodium < 130 mmol/L, a clear dose–response relationship. Gankam Kengne et al. [30] in Belgium gave an adjusted OR of 4.16 (95% CI 2.24–7.71) for fragility fracture, and Kinsella et al. [31] reported an adjusted OR of 2.25 for fracture occurrence, independent of osteoporosis.

Simultaneously, the fracture event and admission themselves generate hyponatraemia. Cumming’s analysis [3,32], often cited as a corrective to SIADH-centric thinking, identified hypovolaemia as the dominant mechanism in 70% of cases, with SIADH accounting for only 27%. The peri-operative incidence data discussed above further confirm the acquired component. The phenotypes are neither mutually exclusive nor always distinguishable at the bedside.

### Falls, Fracture Risk and Bone

The seminal study is Renneboog et al.’s [20] case–control comparison of 122 ambulatory patients with mean sodium 126 ± 5 mmol/L against 244 matched controls. Falls occurred in 21.3% versus 5.3%, with an adjusted odds ratio of 67 (95% CI 7.5–607), clinically illustrative if statistically imprecise. Gait (centre-of-pressure travelled way 1336 vs. 1047 mm, *p* = 0.003) and attention (reaction time 673 vs. 615 ms; 1.2-fold increase in errors) were objectively impaired to a degree comparable to a blood alcohol concentration of approximately 0.6 g/L. Subsequent work by Decaux [21] and Gankam Kengne et al. [30] has confirmed the signal in older adults and in fragility fracture cohorts.

Verbalis and colleagues [22], in a rat SIADH model, showed that chronic hyponatraemia produced substantial bone loss independent of falls: three months of induced hyponatraemia reduced femoral BMD by approximately 30%, with histomorphometry showing an approximately fivefold increase in osteoclast number on trabecular surfaces and reduced bone formation. Concurrent NHANES III analysis demonstrated an adjusted OR of 2.85 (95% CI 1.03–7.86) for osteoporosis of the hip in adults with sodium <135 mmol/L. Human observational data from Kruse et al. [33] in the Danish registry (adjusted OR for total hip osteoporosis 2.17), Holm et al. [34] in 5610 women (increased hazard for major osteoporotic fracture independent of osteoporosis and medication), Usala et al. [35] and Adams et al. [36] are concordant. The 2019 meta-analysis by Murthy and colleagues pooled a fracture OR of 2.34 (95% CI 1.86–2.96) and an osteoporosis OR of 2.67, with a mortality OR of 1.31 [37].

The NHFD does not currently capture serum sodium as a core registry variable; UK prevalence inferences derive from NHFD-linked local cohorts [3,9,16].

## 4. Pathophysiology of Hyponatraemia in Relation to NOF Fracture

### 4.1. Classification and Aetiology in the NOF Patient

The 2014 European guideline and the 2013 US expert panel converge on an aetiological framework anchored by extracellular volume status [1,38]. In NOF patients, hypovolaemic hyponatraemia predominates at admission (diuretic effects, gastrointestinal losses, reduced oral intake, concealed fracture haematoma, and pre-admission immobility), euvolaemic SIADH becomes important peri-operatively (pain, nausea, opioids, surgical stress, medications), and hypervolaemic hyponatraemia occurs in patients with coexisting heart failure, cirrhosis or advanced kidney disease.

### 4.2. SIADH in the Fracture Context

Hip fracture patients experience a convergence of non-osmotic AVP stimuli. Nausea is one of the most potent, capable of raising plasma AVP approximately ten-fold and overriding concurrent osmotic suppression [39]. Pain afferents project to the supraoptic and paraventricular nuclei, where nociceptive signalling directly stimulates magnocellular AVP release; inflammatory cytokines (IL-6, IL-1β) released from the injured bone and soft tissue amplify this response [40,41]. The neuroendocrine stress response of surgery maintains elevated AVP for 3–5 days post-operatively, with plasma levels reported as 20–50-fold above basal in the first 48 h. When hypotonic intravenous fluid is administered during this window, the renal capacity to excrete free water is overwhelmed, which is the mechanism characterised by Moritz and Ayus [42,43] as hospital-acquired hyponatraemia and the basis for their advocacy of isotonic maintenance regimens.

### 4.3. Drug-Induced Hyponatraemia: Mechanisms

Thiazides inhibit the sodium–chloride cotransporter (NCC) in the distal convoluted tubule, impairing urinary dilution while leaving concentrating capacity intact, a paradox that explains why loop diuretics (which disrupt the medullary countercurrent gradient) cause hyponatraemia less readily. Recent genetic work by Ware et al. [44] implicates SLCO2A1/prostaglandin transporter polymorphisms, which augment luminal prostaglandin E2, EP4-mediated aquaporin-2 insertion and free-water retention in susceptible individuals. Volume depletion and intracellular potassium loss further destabilise the osmotic milieu. Risk is highest in the first one to two weeks of therapy.

SSRIs cause a drug-induced SIADH by potentiating hypothalamic serotonergic and α-adrenergic signalling at magnocellular neurons, with secondary upregulation of V2 receptor/aquaporin-2 signalling. Combined diuretic-plus-SSRI therapy confers markedly amplified risk: in the case–control study of Movig and colleagues, concurrent SSRI and diuretic use was associated with an odds ratio for hyponatraemia of 8.4 (95% CI 2.1–34) overall, rising to 13.5 (95% CI 1.8–101) in those aged ≥65 years, although the wide confidence intervals reflect the small subgroup size [45]. Carbamazepine and oxcarbazepine enhance renal responsiveness to vasopressin via the V2-receptor/aquaporin-2 pathway (oxcarbazepine additionally through inhibition of prostaglandin E2-mediated cyclic AMP suppression) rather than acting as direct V2-receptor agonists; reported incidences of hyponatraemia in the literature, as compiled by Berghuis et al. [46], span 4–40% with carbamazepine and 23–73% with oxcarbazepine, varying with age, dose and the sodium threshold applied. Proton pump inhibitors show a robust epidemiological association, most strikingly in the Swedish case–control study of Falhammar et al. [27]. Mechanism remains speculative and may involve secondary hypomagnesaemia. Opioids contribute via multiple pathways: direct μ-receptor stimulation of magnocellular neurons, opioid-induced nausea, AVP-independent antidiuresis (demonstrated in Brattleboro rats), and, in the case of tramadol, serotonergic potentiation of AVP release [47].

### 4.4. Cerebral and Renal Salt Wasting

Cerebral salt wasting is relevant to NOF patients who sustain concurrent head injury at the time of the fall, a more common scenario than routinely documented. Proposed mechanisms centre on brain-derived natriuretic peptides and disrupted renal sympathetic input, producing natriuresis with true hypovolaemia [48,49]. Differentiation from SIADH is treatment-defining (volume replacement rather than fluid restriction), though the existence of cerebral salt wasting as a discrete entity remains contested.

### 4.5. Hypovolaemia Specific to NOF

Three often-underappreciated contributors operate simultaneously. First, concealed blood loss from the fracture haematoma can be substantial: Smith et al. [50] documented a mean pre-operative haemoglobin decline of 20.2 g/L in extracapsular and 14.9 g/L in intracapsular fractures, with some patients losing up to 59 g/L. Foss and Kehlet [51] demonstrated total hidden blood loss of 547–1473 mL, substantially exceeding intra-operative measured loss. Second, pre-operative fasting compounds pre-existing age-related volume contraction in patients with blunted thirst and reduced renal concentrating capacity. Third, the “long lie” phenomenon, prolonged floor time before discovery, produces insensible water loss, reduced intake, often rhabdomyolysis (releasing IL-6 as a further non-osmotic AVP stimulus), and clinically important hypovolaemia.

### 4.6. Bone Pathophysiology: The Mechanistic Crux

That hyponatraemia may cause bone loss independent of falls is arguably the most novel insight of the last fifteen years. Three complementary mechanisms have been proposed; it should be emphasised at the outset that the direct mechanistic evidence derives predominantly from rodent models and in vitro cell systems, and that these pathways remain hypotheses extrapolated to the hip-fracture patient rather than mechanisms demonstrated in that population.

First, bone is a major sodium reservoir. The classical studies of Bergstrom and Wallace [52] established that approximately 30–40% of total body sodium resides in bone, approximately one-third of which is rapidly exchangeable. Chronic extracellular sodium deficit mobilises this reservoir at the cost of mineral matrix integrity.

Second, in cultured cells, low extracellular sodium directly stimulates osteoclastogenesis. Barsony et al. [53] showed that reduced [Na^+^], not reduced osmolality per se, dose-dependently augments osteoclast differentiation and resorptive activity in cultured cells. The mechanism involves impaired function of the sodium-dependent ascorbic acid transporter (SVCT2), reduced intracellular ascorbate uptake, oxidative stress, and upregulation of NF-κB/NFATc1 signalling with elevated RANKL/OPG ratios. Transcriptomic analysis confirms osteoclasts as primary cellular responders [54].

Third, in experimental models, AVP itself acts directly on bone. Tamma et al. [55] demonstrated AVPR1α and AVPR2 expression on both osteoblasts and osteoclasts, and showed that pharmacological or genetic inhibition of AVPR1α increases bone mass in mice. High AVP, the common currency of SIADH and hypovolaemic non-osmotic states (both characteristic of NOF), therefore stimulates bone resorption and inhibits formation directly, synergising with the cellular low-sodium effect.

**Figure 1:**
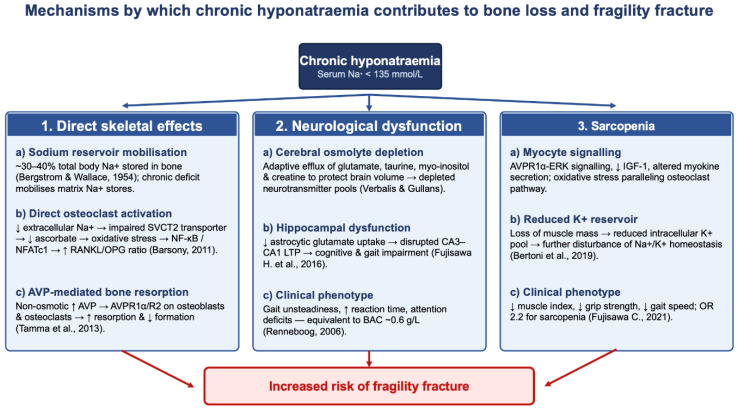
Three convergent mechanisms by which chronic hyponatraemia contributes to bone loss and increased fragility fracture risk: direct skeletal effects (sodium reservoir mobilisation, osteoclastogenesis and AVP-mediated bone resorption), neurological dysfunction producing falls, and sarcopenia. AVP, arginine vasopressin; BAC, blood alcohol concentration; BMD, bone mineral density; LTP, long-term potentiation; NFATc1, nuclear factor of activated T-cells, cytoplasmic 1; OPG, osteoprotegerin; RANKL, receptor activator of nuclear factor-κB ligand; SVCT2, sodium-dependent vitamin C transporter 2. Sources: [20,52,53,55,57,58,59,60].

In the aged rat, 18 weeks of chronic hyponatraemia produce progressive BMD loss, reduced bone sodium and calcium content, hypogonadism, cardiomyopathy and skeletal muscle sarcopenia, a composite phenotype remarkably similar to human frailty and partially attenuated by vitamin D supplementation [56]. These proposed mechanisms are summarised in Figure 1.

### 4.7. Neurological Basis of Falls

Neurological adaptation to chronic hyponatraemia is characterised by two phases: rapid efflux of intracellular electrolytes (minutes to hours) and slow efflux of organic osmolytes, glutamate, taurine, myo-inositol, glutamine, glycine and creatine, over hours to days [57,61]. Brain volume is thereby protected at the cost of depletion of neurotransmitter pools. Fujisawa H. et al. [58] demonstrated that chronic hyponatraemia elevates extracellular hippocampal glutamate, impairs astrocytic glutamate uptake, disrupts CA3–CA1 long-term potentiation, and produces reversible gait disturbance (smaller stride, wider stance) and cognitive impairment. Clinically, these molecular changes manifest as the gait unsteadiness, prolonged reaction time and attention deficits quantified by Renneboog et al. [20]. The phenotype is clinically silent by conventional consciousness measures yet functionally equivalent to mild alcohol intoxication, explaining why “asymptomatic” hyponatraemia measurably increases fall risk.

### 4.8. Sarcopenia

Animal and human data converge on a sarcopenic phenotype. Barsony et al.’s [56] aged rat model shows measurable thigh lean-mass loss. In humans, Fujisawa C. et al. [59] reported that elderly patients with mild hyponatraemia (Na 130–135 mmol/L) had lower skeletal muscle index, weaker grip strength (19.1 vs. 21.4 kg) and slower gait (0.9 vs. 1.1 m/s), with an independent OR of 2.2 for sarcopenia. The postulated mechanism combines AVPR1α-ERK signalling in myocytes, oxidative stress paralleling the osteoclast mechanism, reduced IGF-1 signalling and altered myokine secretion. The bidirectional hypothesis, that reduced muscle mass reduces intracellular potassium reservoirs and thereby disturbs sodium–potassium homeostasis, has also been advanced [60]. A vicious cycle has been proposed, in which hyponatraemia may drive sarcopenia and osteopenia, which together increase fall risk and fracture susceptibility, and the fracture itself then precipitates further hyponatraemia.

## 5. Impacts of Hyponatraemia with NOF in Main Clinical Outcomes

The estimates in Table 1 are drawn from individual studies that differ substantially in design, case mix, sodium thresholds, timing of measurement and covariate adjustment; the figures are therefore best read as illustrative of the direction and approximate magnitude of effect within each cited cohort rather than as pooled or directly comparable values, and the ~1.15–1.40 adjusted 30-day mortality range should be interpreted as a summary of separate point estimates across heterogeneous cohorts rather than a meta-analytic result. With that caveat, the evidence summarised in Table 1 paints a consistent but nuanced picture. Three signals are reproducible across cohorts: hyponatraemia at admission is associated with a modest but real excess in short-term mortality (adjusted hazard ratios ~1.15–1.40), a 30–50% prolongation of hospital stay, and a measurable delay to surgery [8,9,23,62]. A clear dose–response gradient is also evident; mortality risk rises stepwise from mild to moderate/severe hyponatraemia [63,64]. By contrast, the picture is less uniform for some outcomes: the EuSOS analysis of mixed surgical patients found no independent mortality signal for hyponatraemia after adjustment, in contrast to the consistent NOF-specific cohort data [65]; readmission rates show no association in either the NOF cohort (30-day) or the arthroplasty cohort (90-day) [17,66]; and functional recovery outcomes remain inadequately studied [62]. A particularly informative contrast comes from Ayus et al. [66]: chronic uncorrected hyponatraemia carried long-term mortality risk, while recent peri-operative hyponatraemia conferred excess post-operative sepsis, suggesting distinct prognostic signatures. Importantly, pre-operative correction has not been shown to improve outcomes; Madsen et al. [8] reported similar 30-day mortality in patients whose hyponatraemia normalised versus persisted, and Aqil et al.’s [4] NHFD analysis demonstrated that hyponatraemia predicts surgical delay without independently predicting outcome, although broader inpatient meta-analyses suggest that hyponatraemia improvement is associated with reduced mortality [67]. On current observational evidence, the clinical inference is that mild isolated hyponatraemia should not by itself delay surgery, although this has not been tested in a randomised trial.

## 6. Critical Appraisal of the Individual Studies and Reasons for Caution

The studies underpinning this review differ considerably in design and robustness, and these differences bear directly on how far each finding can be used as evidence-based and applied to neck of femur fracture patients. Two features are common to almost all of them and are the principal reason for caution: every human study cited here is observational, and no interventional trial has been completed, so no study can show that hyponatraemia causes fracture, poor surgical outcomes or death rather than marking the frailty and comorbidity with which low sodium travels. The specific weakness of each principal study is set out below.

The prevalence figures rest largely on registry and single-centre data. The Danish national registry analysis of Madsen et al. [8] and the Tayside cohort of Tinning et al. [9] included large numbers, but records of this kind capture serum sodium at varying times; rarely document the cause of hyponatraemia, medication exposure or volume status; and remain open to residual confounding, so their prevalence and mortality estimates are best read as associations within a defined dataset rather than as causal or fully generalisable values. The Aberdeen cohort of Cumming et al. [3], although prospective, studied a mixed fragility fracture group of whom only about half had a proximal femoral fracture, so its figures are only partly specific to neck of femur fracture, and, as already noted, its admission and in-hospital rates are reported separately without the overlap being specified, making their sum an upper bound rather than a measured prevalence. The single-centre and regional series from Italy [10,11], Japan [12] and the United States [13,14] are small, apply differing sodium thresholds and, where case–control in design, are open to selection effects, which is why their estimates scatter rather than agree closely. The meta-analysis of Corona et al. [15] pools fifteen such observational studies, so although it produces a precise-looking odds ratio of 2.00, it inherits their confounding and heterogeneity and cannot overcome their observational nature.

Estimates of acquired, post-operative hyponatraemia come from small or non-specific work. Rudge and Kim [16] studied only 254 operated patients at a single UK centre, and Hennrikus et al. [24] reported on general orthopaedic rather than neck of femur patients, so both indicate the scale of the problem but offer limited precision and generalisability.

The evidence that chronic hyponatraemia precedes and predicts fracture is consistent in direction but uniformly observational. The Rotterdam study [2] and the MrOS study [28] are community cohorts rather than fracture surgery populations, and MrOS enrolled only men; both adjusted for bone mineral density and falls but cannot exclude unmeasured confounding by frailty and comorbidity. The large analysis of Ayus et al. [29] reports a striking hazard ratio for hip fracture, but its wide confidence interval, from 2.14 to 9.60, signals imprecision, and it too is observational. The studies of Gankam Kengne et al. [30] and Kinsella et al. [31] are smaller observational analyses that share the same susceptibility to confounding.

The mechanistic and falls literature is the most striking but the least directly applicable to hip fracture patients. The seminal study of Renneboog et al. [20] included only 122 hyponatraemic patients, compared with 244 controls, and produced an adjusted odds ratio for falls of 67, with a confidence interval from 7.5 to 607; as the review already states, this is statistically imprecise and should be read as illustrative rather than a reliable effect size. Much of the causal mechanism rests on animal and cell work, including the rat model of Verbalis et al. [22], the cultured-cell studies of Barsony et al. [53,54], the mouse work of Tamma et al. [55], the aged-rat model of Barsony et al. [56] and the rodent model of Fujisawa H. et al. [58]; these demonstrate biologically plausible pathways, but effects seen in rodents and cell systems cannot be assumed to hold, or to hold at the same magnitude, in older humans with a fracture. The human osteoporosis and fracture associations of Kruse et al. [33]; Holm et al. [34], who studied women only; Usala et al. [35]; and Adams et al. [36] are cross-sectional or registry-based and so cannot separate low sodium from the conditions that accompany it, while the meta-analysis of Murthy et al. [37] again pools observational studies of differing quality. The NHANES III association reported alongside Verbalis et al. [22] is cross-sectional, and its confidence interval, from 1.03 to 7.86, has a lower bound close to no effect.

The sarcopenia evidence is limited and partly hypothetical. The human study of Fujisawa C. et al. [59] is cross-sectional and modest in size, so it cannot show that hyponatraemia causes muscle loss, and the muscle-to-sodium mechanism proposed by Bertoni et al. [60] is explicitly a hypothesis rather than a demonstrated pathway.

The outcome associations, though reproducible in direction, come from heterogeneous observational studies that, as Table 1 already cautions, should not be pooled or read as directly comparable. The meta-analysis of Teo et al. [23] combines thirty-two observational studies in mixed surgical populations rather than in neck of femur fracture specifically. The post-operative sepsis signal from Ayus et al. [66] rests on an odds ratio of 1.84 whose confidence interval, from 1.01 to 3.35, barely excludes no effect, and its distinction between chronic and recent hyponatraemia, while biologically plausible, has not been replicated in other neck of femur cohorts. The prognosis data of Cumming et al. [62] come from a small cohort, and the cohort of Cunningham et al. [17] describes elective arthroplasty rather than fracture surgery. Aqil et al. [4] show that hyponatraemia predicts surgical delay without independently predicting outcome, and the EuSOS analysis of Cecconi et al. [65] found no independent mortality signal after adjustment in mixed surgical patients, a discordance that is itself a reason for caution. Most importantly, the only direct evidence on treatment, from Madsen et al. [8], shows no mortality difference between patients whose hyponatraemia corrected and those in whom it persisted, so no benefit of correction can be claimed.

The drug-association studies carry further caveats. The pharmacogenetic findings of Ware et al. [44] apply to susceptible individuals rather than to patients generally; the antidepressant case-control study of Movig et al. [45] rests on small subgroups with very wide confidence intervals, as the review notes; the carbamazepine and oxcarbazepine figures compiled by Berghuis et al. [46] derive from people with epilepsy rather than fracture patients and span a very wide range; and the proton pump inhibitor association of Falhammar et al. [27] comes from a case–control design whose original estimate was later revised in a corrigendum.

Taken together, these limitations do not remove the consistency of the associations, which is real and reproducible, but they do mean that the direction and rough size of effect are far better supported than any claim of cause. This is why the guidance in this review is deliberately conservative and confined to what observational data can bear, namely not delaying surgery for mild isolated hyponatraemia, while active correction to improve fracture or surgical outcomes awaits the interventional evidence that alone could justify it.

## 7. Discussion and Synthesis

The available data are consistent with chronic mild hyponatraemia being more than a passive biomarker and are compatible with a causal contribution to fracture risk through parallel bone, neurological and muscular pathways [2,22,29,53,55], but they cannot establish that the relationship is causal. This interpretation must, however, be made cautiously: the supporting human evidence is almost entirely observational and therefore susceptible to residual confounding. Hyponatraemia in this population travels with frailty, multimorbidity, heart failure, chronic kidney disease, systemic inflammation, volume-status abnormalities, malnutrition and polypharmacy, each of which is independently associated with both low sodium and adverse bone and mortality outcomes; the persistence of associations after adjustment in several cohorts is reassuring but cannot exclude residual or unmeasured confounding, and no interventional data establish causality in humans.

The clinical practice implication most directly supported by evidence is not to delay surgery for mild isolated hyponatraemia. The NHFD-derived analysis of Aqil et al. [4] demonstrates that hyponatraemia predicts delay without predicting outcome. Given the well-established mortality penalty of delay beyond 36–48 h in hip fracture, the practical inference is: electrolyte derangement of mild degree should prompt investigation and cautious correction alongside, not prior to, timely surgery. Because no interventional study has tested pre-operative correction, this guidance rests on observational data alone, and no active correction strategy can currently be recommended to improve fracture or surgical outcomes.

The mechanistic picture has transformed from a homogeneous SIADH-centric model to a heterogeneous, hypovolaemia-dominated phenotype. Cumming’s identification of SIADH in only 27% of cases, with dehydration in 70% and thiazide use in 76% [3], may suggest the need to recalibrate the reflexive use of fluid restriction, which risks worsening hypovolaemic hyponatraemia and compromising surgical optics.

The chronic–acute dichotomy within hyponatraemia outcomes warrants formal study. Ayus et al.’s [66] finding that chronic prolonged hyponatraemia predicts long-term mortality while recent hyponatraemia predicts acute sepsis is biologically plausible and clinically actionable, but has not been replicated in other NOF cohorts. It is also of interest to assess the impact of osmstat reset hyponatremia (regarded as normal condition in large numbers of the elderly population) on the risk of bone fracture.

A peculiar gap exists around functional and rehabilitation outcomes, and this can be addressed in future retrospective studies.

Limitations of the existing evidence include heterogeneity of sodium thresholds; near-universal reliance on observational designs vulnerable to residual confounding by frailty, multimorbidity, heart failure, chronic kidney disease, inflammation, volume-status abnormalities, malnutrition and polypharmacy; inconsistent reporting of severity strata and trajectory; and minimal attention to classical endocrine causes (hypothyroidism, adrenal insufficiency) in NOF-specific cohorts. Much of the mechanistic literature is derived from animal or in vitro models and has not been confirmed in patients with hip fracture. This review is narrative, and the individual studies are appraised descriptively in the critical appraisal rather than with a numerical scoring system, so the strength of each association should be interpreted in light of the study-specific weaknesses set out there. No randomised controlled trial of pre-operative sodium correction in NOF has been completed, and no NOF-only outcome meta-analysis exists.

## 8. Conclusions

Hyponatraemia is the commonest electrolyte disturbance in neck of femur fracture, present in roughly one in five patients at admission and developing in a further one in five to one in three during hospitalisation. It is both a consequence of the fracture event, through hypovolaemia, concealed blood loss, pain-driven AVP release and iatrogenic fluid administration, and, on the balance of observational and mechanistic evidence, possibly a contributor to fracture risk, through three postulated pathways that remain unproven as causal mechanisms in humans: sodium- and AVP-dependent bone resorption, subtle neurological dysfunction producing falls, and sarcopenia. Whether these associations are wholly or only partly causal remains unresolved, as the human evidence is observational and confounded by frailty and its correlates. Every study underpinning these associations is observational or preclinical and carries specific methodological weaknesses, set out one by one in the critical appraisal, so each association is a reason for caution rather than proof of effect, and no interventional evidence exists to confirm that treating hyponatraemia changes fracture or surgical outcomes. It is consistently associated with longer hospital stay, delayed surgery and a ~1.15–1.40-fold adjusted 30-day mortality hazard, with a clear severity gradient. Pre-operative correction has not been shown to improve outcomes, and surgery should not be delayed for mild isolated hyponatraemia.

Priority research directions include: (i) a randomised controlled trial of structured pre-operative correction versus expedited surgery in mild to moderate hyponatraemia; (ii) prospective stratified evaluation of rehabilitation outcomes and return to pre-fracture function by admission natraemia; (iii) mechanistic human studies quantifying the contribution of AVP-driven bone resorption in the acute peri-fracture window; and (iv) interventional studies of correction strategies, including vaptans or targeted anti-resorptive therapy, in chronic hyponatraemic older adults at high fracture risk, noting that, as of 2026, no vaptan trial with bone mineral density or fracture endpoints has been registered or reported. Until such evidence emerges, the most defensible clinical stance is one of vigilance without procrastination, recognising hyponatraemia as a marker of generalised frailty and proceeding to surgery promptly while addressing the electrolyte disturbance in parallel.

## Figures and Tables

**Table 1:** Summarises the principal clinical outcome associations of hyponatraemia in NOF fracture, with key effect estimates and source studies. The narrative below provides supporting context for each domain.

Outcome	Effect Estimate	Population/Source	Notes
30-day mortality	aHR 1.38 (95% CI 1.16–1.64); 12.2% vs. 9.6% (*p* = 0.005)	Danish national registry, *n* = 7317	[8]
Long-term survival	aHR 1.15 for admission and post-op hyponatraemia; median survival 34 vs. 41 months	Tayside cohort, *n* = 3897	[9]
Composite mortality (surgical)	aHR 1.27 (95% CI 1.13–1.43); aHR 1.32 even for mild	Meta-analysis, *n* ≈ 1.3 million	[23]
Major post-operative complications	aOR 1.37 (95% CI 1.23–1.53)	Meta-analysis [23]	Respiratory, renal, septic events
Post-operative sepsis	aOR 1.84 (95% CI 1.01–3.35)	Argentine NOF cohort, *n* = 1571	[66], recent only
Length of stay	+51.5% LOS (*p* = 0.006); 30 vs. 21 days for moderate post-op	[16,62]	Consistent across NOF cohorts
Time to surgery	+66.7% (*p* = 0.014); aRR 1.24 for delay >36 h (95% CI 1.06–1.44)	[4,62]	Hyponatraemia: only significant medical cause of delay
Severity gradient (mortality)	Mild aHR 1.80 → moderate/severe aHR 2.47	[63]	Clear dose–response
30-day readmission	No association	[17,66]	Both chronic and recent
Functional recovery	Trend toward inpatient rehabilitation (OR 2.2, NS)	[62]	Significant evidence gap
Effect of correction	30-day mortality 10.4% (corrected) vs.11.3% (persistent), *p* = 0.6	[8]	Pre-op correction not associated with improved outcomes

Summary of clinical outcome associations of hyponatraemia in neck of femur fracture. Estimates derive from individual studies with differing designs, populations, sodium thresholds and adjustment, and are not pooled; they should not be read as directly comparable. aHR, adjusted hazard ratio; aOR, adjusted odds ratio; aRR, adjusted relative risk; CI, confidence interval; LOS, length of stay; NS, not significant.

## Data Availability

No new data were created or analysed in this study. Data sharing is not applicable to this article.
